# Ratio of lymphocyte to monocyte area under the curve as a novel predictive factor for severe infection in multiple sclerosis

**DOI:** 10.3389/fimmu.2023.1133444

**Published:** 2023-02-14

**Authors:** Junichiro Takahashi, Tomoko Okamoto, Youwei Lin, Reiko Saika, Atsuko Katsumoto, Wakiro Sato, Takashi Yamamura, Yuji Takahashi

**Affiliations:** ^1^ Department of Neurology, National Center Hospital, National Center of Neurology and Psychiatry, Kodaira, Tokyo, Japan; ^2^ Department of Immunology, National Institute of Neuroscience, National Center of Neurology and Psychiatry, Kodaira, Tokyo, Japan

**Keywords:** lymphocyte, monocyte, area under the curve, infection, predictive factor

## Abstract

**Background:**

Individuals with multiple sclerosis (MS) are vulnerable to all types of infection, because MS itself involves immunodeficiency, in addition to involving treatment with immunosuppressants. Simple predictive variables for infection that are easily assessed in daily examinations are warranted. Lymphocyte area under the curve (L_AUC), defined as the sum of serial absolute lymphocyte counts under the lymphocyte count-time curve, has been established as a predictive factor for several infections after allogenic hematopoietic stem cell transplantation. We assessed whether L_AUC could also be a useful factor for predicting severe infection in MS patients.

**Methods:**

From October 2010 to January 2022, MS patients, diagnosed based on the 2017 McDonald criteria, were retrospectively reviewed. We extracted patients with infection requiring hospitalization (IRH) from medical records and matched with controls in a 1:2 ratio. Variables including clinical severity and laboratory data were compared between the infection group and controls. L_AUC was calculated along with the AUC of total white blood cells (W_AUC), neutrophils (N_AUC), lymphocytes (L_AUC), and monocytes (M_AUC). To correct for different times of blood examination and extract mean values of AUC per time point, we divided the AUC by follow-up duration. For example, in evaluating lymphocyte counts, we defined the ratio of [L_AUC] to [follow-up duration] as [L_AUC/t]. Multivariate regression analysis was conducted to extract predictive factors associated with IRH. Also, discriminative analysis was conducted using candidate variables from multivariate analysis.

**Results:**

The total case-control sample included 177 patients of MS with IRH (n=59) and non-IRH (controls) (n=118). Adjusted odds ratios (OR) for the risk of serious infection in patients with MS with higher baseline expanded disability status scale (EDSS) (OR 1.340, 95% confidence interval [CI] 1.070–1.670, *p* = 0.010) and lower ratio of L_AUC/t to M_AUC/t (OR 0.766, 95%CI 0.591–0.993, *p* = 0.046) were significant. Notably, the kind of treatment, including glucocorticoids (GCs), disease-modifying drugs (DMDs) and other immunosuppressants agents, and dose of GCs were not significantly associated with serious infection after correlated with EDSS and ratio of L_AUC/t to M_AUC/t. In discriminative analysis, sensitivity was 88.1% (95%CI 76.5–94.7%) and specificity was 35.6% (95%CI 27.1–45.0%), using EDSS ≥ 6.0 or ratio of L_AUC/t to M_AUC/t ≤ 3.699, while sensitivity was 55.9% (95%CI 42.5–68.6%) and specificity was 83.9% (95%CI 75.7–89.8%), using both EDSS ≥ 6.0 and ratio of L_AUC/t to M_AUC/t ≤ 3.699.

**Conclusion:**

Our study revealed the impact of the ratio L_AUC/t to M_AUC/t as a novel prognostic factor for IRH. Clinicians should pay more attention to laboratory data such as lymphocyte or monocyte counts itself, directly presenting individual immunodeficiency, rather than the kind of drug to prevent infection as a clinical manifestation.

## 1 Introduction

Multiple sclerosis (MS) is an autoimmune-mediated demyelinating disorder of the central nervous system (1) that negatively influences physical disability in young adults. MS itself may be caused by an overactive immune system that attacks the protective layer of the nerves in the brain. As a result of this overactivity, the immune system is not able to fight infections as normal, resulting in a higher risk of infection-associated hospitalization (2, 3) or even death from infection (4–6) compared to the general population. All types of infection are increased in patients with MS: viral, fungal, bacterial, and also opportunistic infection. In addition, most patients are treated with immunosuppressants, including disease-modifying drugs (DMDs) and glucocorticoids (GCs), increasing the risk of infection. Infection itself has a critical influence on MS patients, not only in terms of infection damage, but also in residual function, because infection can worsen MS symptoms, especially in patients with higher levels of disability (7). Clinicians should thus pay more attention to preventing infections rather than treating clinical manifestations. For this purpose, simple predictive variables that are easily assessed in daily examinations are needed. We assessed a new biomarker, lymphocyte area under the curve (L_AUC), as a new predictive factor for infection by evaluating its impact on severe infection.

L_AUC is defined as the sum of serial absolute lymphocyte counts under the lymphocyte count-time curve (8). This value has been established as a predictor of several infections after allogenic hematopoietic stem cell transplantation (9). Infection risk could be more closely associated with L_AUC than with absolute lymphocyte counts as determined at a single time point, because L_AUC better reflects the duration and severity of lymphocytopenia. Our aim was to evaluate predictors of IRH in MS patients and assessed the impact of L_AUC as a simple surrogate marker for IRH.

## 2 Methods

### 2.1 Patients

Medical records of patients with MS, diagnosed based on the 2017 McDonald criteria, at the National Center Hospital, National Center of Neurology and Psychiatry were retrospectively reviewed. Participants in this study comprised MS patients without acute relapses, defined as neurological episodes of more than 24 h and separated from a previous attack by at least 30 days, seen at the National Center Hospital from October 2010 to January 2022.

### 2.2 Study design

The study was conducted in a single center using a retrospective observational case-control design.

The definition of infection focused on the requirement for hospitalization, because of the difficulty of following-up patients on transient therapies such as oral anti-macrobiotics prescribed during outpatient visits. Patients with MS were observed from the date of first visit to our hospital as the earliest incidence of infection until death, latest hospital visit, or the end of the study period (January 11, 2022), whichever occurred first. Patients taking investigational drugs during follow-up or with missing clinical data were excluded. The study used a case-control design within a cohort to investigate associations between diverse clinical characteristics, including blood examinations. Among patients with MS in the study hospital, patients with IRH were matched to MS patients without infection as controls. IRH was identified using the following criteria: 1) the infection date was the date on which antibiotics were first prescribed, and considered as the index date; 2) period of antibiotic use >3 days; and 3) infections identified within 7 days after antibiotic prescription were considered as the same infection.

**Figure 1:**
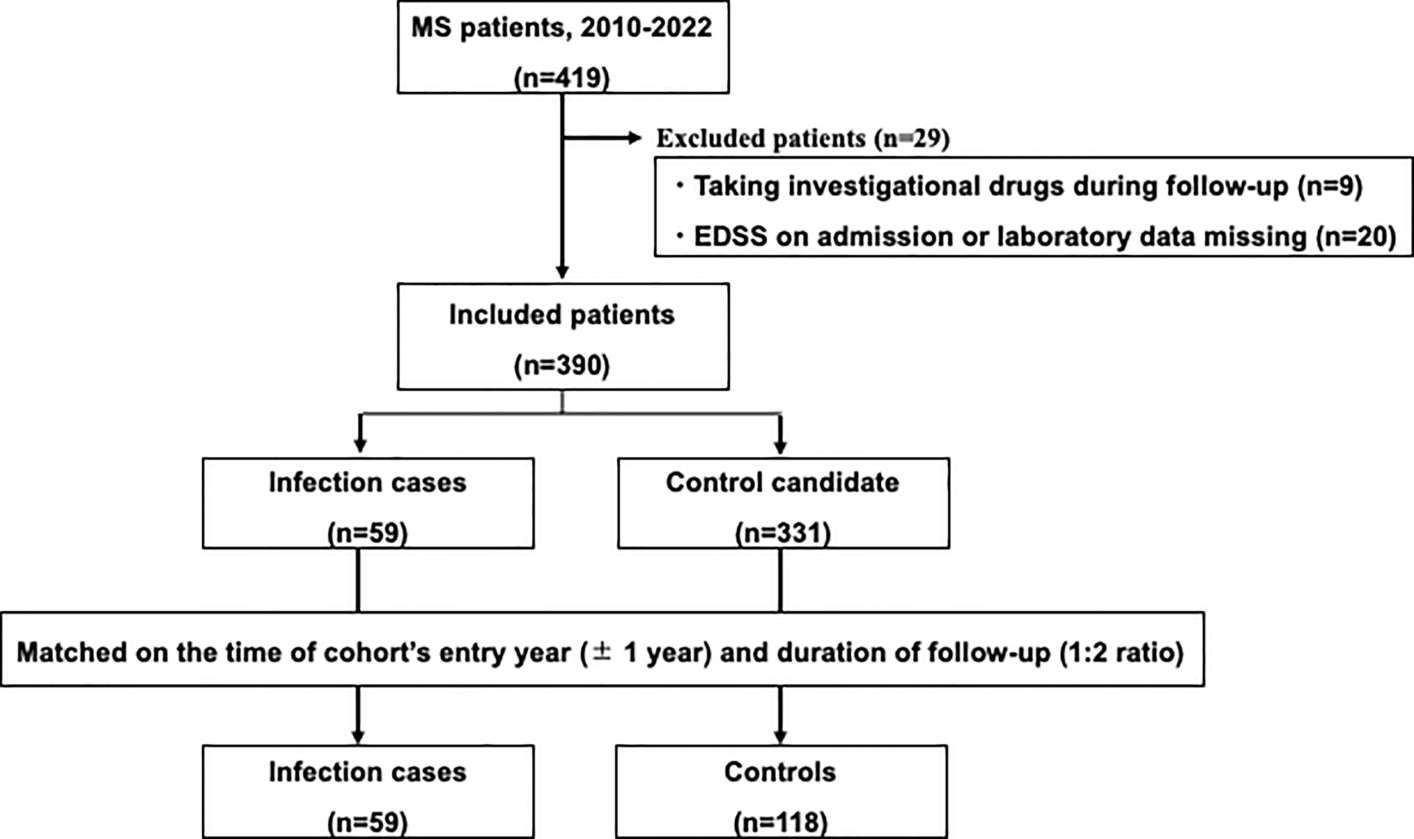
Study flow chart. Among patients with multiple sclerosis in the study hospital, patients with serious infections needing hospitalization were matched with controls without serious infections.

Controls were randomly selected from MS patients without infection after matching for the time of entry year (± 1 year) and duration of follow-up. The 59 infection cases were individually matched in a 1:2 ratio with 118 controls (**Figure 1**).

### 2.3 Site of infection

All infections were categorized as bacterial (upper respiratory tract infection, pneumonia, urinary tract infection, gastrointestinal tract infection, cellulitis, bacteremia), fungal (pneumonia, vaginitis), or viral (upper respiratory tract infection, pneumonia, antigenemia, enteritis, dermatitis, myelitis, progressive multifocal leukoencephalopathy).

### 2.4 Area under the curve of leukocytes

Blood examinations were conducted at least twice within a 3-month period. We calculated each AUC for total white blood cells (W_AUC), neutrophils (N_AUC), lymphocytes (L_AUC), and monocytes (M_AUC) for 6 months before infection onset for infection group or from the baseline point for controls. Also, to correct for the different times of blood examinations and extract the mean value of AUC per time point, we divided AUC by the follow-up duration. For example, in the evaluation of lymphocyte counts, we defined the ratio of [L_AUC] to [follow-up duration] as [L_AUC/t].

### 2.5 Data collection

Variables identified for analysis as potential contributors to infection risk included sociodemographic variables (age at initial symptom, sex, type of multiple sclerosis as relapsing-remitting, secondary progressive, or primary progressive), clinical status (age on infection or baseline, clinical duration, EDSS at baseline), and blood examination (W_AUC, N_AUC, L_AUC, M_AUC). In terms of treatment details, DMDs (interferon-beta 1a/1b, glatiramer acetate, dimethyl fumarate, fingolimod, natalizumab, siponimod fumaric acid, and ofatumumab), steroids with doses converted to prednisolone-equivalents (prednisolone 5 mg = methylprednisolone 4 mg = hydrocortisone 20 mg), and other immunosuppressants (azathioprine, methotrexate, bucillamine, tacrolimus, cyclosporine), and duration of drug use were assessed. We also defined the mean GC dose as the average dose across the prescription period. Cumulative dose of intravenous methylprednisolone (IVMP) for 6 months before infection or from baseline was also calculated. All baseline demographic data were obtained from medical records.

### 2.6 Statistical analysis

Descriptive statistics were used to analyze patient characteristics, laboratory test results, and medication use in IRH patients and matched controls. Groups were matched according to the duration of follow-up and year of cohort entry. Multivariate logistic regression analysis was conducted to estimate odds ratio (OR) and 95% confidence interval (CI) to assess associations between clinical characteristics and serious infection in patients with MS. Variables showing *p* < 0.05 in univariate analyses were selected as candidate variables for multivariable analysis. Values of *p* < 0.05 were considered statistically significant. All statistical analyses were performed with EZR (Saitama Medical Center, Jichi Medical University, Saitama, Japan), a graphical user interface for R version 4.1.1 (R Foundation for Statistical Computing, Vienna, Austria).

### 2.7 Ethics approval and consent to participate

This study was approved and the need to obtain informed consent was waived by the institutional review board at the National Center Hospital, National Center of Neurology and Psychiatry, because the anonymized data were obtained retrospectively.

## 3 Results

### 3.1 Characteristics of infection

Among the total of 59 cases with infection, total number of infections were 68, because some patients developed multiple sites of infection. Sixty (88%) of the 68 infections were bacterial, comprising 5 (7%) upper respiratory tract infections, 11 (16%) cases of pneumonia, 21 (31%) urinary tract infections, 2 (3%) gastrointestinal tract infections, 16 (24%) cases of cellulitis, and 5 (7%) cases of bacteremia (**Table 1**).

**Table 1:** Origins of infection and pathogens.

Origin of Infection	N	Pathogens
Bacterial infection	60	
URI	5	*Haemophilus influenza* (3), *alpha-hemplytic streptococcus* (1), *not identified* (2).
Pneumonia	11	*Mycoplasma pneumoniae* (3), *Hemophilis influenza* (3), *Pseudomonas aeruginosa* (2), *Neisseria species* (1), *Streptococcus pneumoniae PISP* (1), *not identified* (2).
UTI	21	*Escherichia coli* (8), *Enterococcus faecalis* (4) , *Pseudomonas aeruginosa* (4), *Klebsiella pneumoniae* (3), *Corynebacterium amycolatum* (1), *Morganella morganii* (1), *Proteus mirabillis ESBL* (1), *Klebsiella aerogens* (1), *Klebsieela oxytoca* (1), *Corynebacterium koseri* (1), *a-hemolytic streptococcus* (1), *not identified* (5).
GI tract infection	2	*Streptococcus anginosus* (1; appenditis/peritonitis), *Eggerthella lenta* (1; appenditis/peritonitis), *Anaerobic GNR* (1; appenditis/peritonitis), *not identified* (1; cholecystitis and enteritis).
Cellulitis	16	*Streptococcus aureus* (2), *Finegoldia magna* (1), *Peptoniphilus asaccharolyticus* (1), *not identified* (12).
Bacteremia	5	*Pseudomonas aeruginosa* (2), *Enterococcus faecalis* (1), *Escherichia coli* (2), *Klebsiella pneumoniae* (1).
Fungal infection	3	*Pneumocystis jiroveccii* (2; pneumonia), *Trichospolin vaginalis* (1; vaginitis).
Viral infection	5	*Influenza virus* (1; bronchitis, 1; pneumoniae), *Cytomegalo virus* (1; pneumoniae/antigenemia, 1;pneumoniae/enteritis), *Varicella-Zoster virus* (1; dermatitis/myelitis), *JC virus* (1; PML)

URI, upper respiratory infection; UTI, urinary tract infection; GI, gastrointestinal; PISP, penicillin-intermediate resistant Streptococcus pneumoniae; ESBL, extended spectrum ß-lactamases; GNR, gram-positive rod; PML, progressive multifocal leukoencephalopathy.

Urinary tract infection (UTI) was the most commonly observed infection. *Escherichia coli, Enterococcus faecalis*, *Pseudomonas aeruginosa*, and *Klebsiella pneumoniae* were frequently confirmed as the causative pathogens for UTI. Cellulitis showed the second highest frequency of infection, but in most cases (12 infections, 75%) no causative pathogen was confirmed. Pneumonia was the next most common, with *Mycoplasma pneumoniae*, *Haemophilus influenzae*, and *Pseudomonas aeruginosa* frequently identified as causative pathogens. Of the 11 cases of pneumonia, 21 cases of UTI, and 16 cases of cellulitis, five developed bacteremia; due to *Pseudomonas aeruginosa* in 2 cases, *Enterococcus faecalis* in 1 case, *Escherichia coli* in 2 cases, and *Klebsiella pneumoniae* in 1 case. As fungal and viral infections, two patients showed infection by *Pneumocystis jirovecii*, two by *Influenza virus*, one by *Cytomegalovirus*, one by *Varicella Zoster Viru* (VZV), and one by *John Cunningham virus*.

### 3.2 Baseline characteristics

**Table 2** displays the general characteristics of the infection and control groups. The total case-control sample included 177 patients with MS admitted between 2010 and 2022. Each infection case (n=59) was matched with two control patients (n=118). The matching variables of follow-up duration and year of entry were distributed evenly between groups.

**Table 2:** Clinical variables between infection cases and controls.

Charactersitcs	All	Infection cases	Controls	*p*
	n=177	n=59	n=118	
Sociodemographic				
Age at initial symptom, years, median (IQR)	33 (26-41)	31 (25-40)	34 (27-41)	0.354
Gender, male, no, (%)	75 (42)	29 (49)	46 (39)	0.202
Type of MS, no, (%)				< 0.001
RRMS	98 (55)	22 (37)	76 (64)	
SPMS	74 (42)	36 (61)	38 (32)	
PPMS	5 (3)	1 (2)	4 (3)	
Clinical status				
Age on admission, years, median (IQR)	48 (39-56)	50 (42-60)	47 (38-55z9	0.073
Clinical duration, years, median (IQR)	13 (7-20)	22 (16-45)	18 (11-45)	0.007
Baseline EDSS, median (IQR)	5.0 (3.0-7.0)	7.0 (6.0-7.5)	4.0 (2.0-6.0)	0.007
Follow-up duration, years, median (IQR)	4.72 (1.77-8.04)	4.49 (1.60-7.57)	5.18 (1.93-8.14)	0.536
Cohort entry, year				0.574
2010-2012	119 (67)	43 (73)	76 (64)	
2013-2015	31 (18)	7 (12)	24 (20)	
2016-2018	9 (5)	3 (5)	6 (5)	
2019-2021	18 (10)	6 (10)	12 (10)	
Treatment agents				
GCs, no, (%)	112 (63)	42 (71)	70 (59)	0.168
Mean dose of GCs, mg/d*	2.5 (0-7.5)	5.0 (0-10.5)	2.5 (0-5.0)	0.005
Total dose of IVMP within 6 months, g	0 (0-3)	0 (0-6)	0 (0-1)	0.006
Immunosuprresant agents, no, (%)				
Azathioprine	21 (12)	11 (19)	10 (9)	0.084
Methotrexate	15 (8)	8 (14)	7 (6)	0.095
Bucillamine	7 (4)	4 (7)	3 (3)	0.340
Tacrolimus	12 (7)	7 (12)	5 (4)	0.113
Cyclosporine	3 (2)	3 (5)	0 (0)	0.064
DMDs, no, (%)				
Interferon-beta 1a/1b	17 (10)	5 (9)	12 (10)	0.794
Glatiramer acetate	13 (7)	6 (10)	7 (6)	0.363
Dimethyl fumarate	18 (10)	4 (7)	14 (12)	0.183
Fingolimod	15 (8)	7 (12)	8 (7)	0.264
Natalizumab	17 (10)	10 (17)	7 (6)	0.053
Siponimod fumaric acid	8 (5)	1 (2)	7 (6)	0.272
Ofatumumab	5 (3)	0 (0)	5 (4)	0.171
Duration using DMDs, years, median (IQR)	0.07 (0.00-1.75)	0.07 (0.00-2.55)	0.04 (0.00-1.52)	0.563

Chi-square test, the Mann-Whitney U test were used for comparison. MS, Multiple sclerosis; RRMS, Relapsing-remitting MS; SPMS, Secondary progressive MS; PPMS, Primary progressive MS; EDSS, Expanded Disability Status Scale; PSL, Prednisolone; GCs, Glucocorticoids; IVMP, Intravenous methylprednisolone; DMDs, Disease modifying drugs. *Prednisolone-equivalent.

Median duration of follow-up did not differ between infection (median, 4.49 years; range, 1.60–7.57 years) and control groups (median, 5.18 years; range, 1.93–8.14 years). In terms of sociodemographic characteristics, age at initial symptoms and frequency of male sex did not differ significantly between groups. Types of MS differed, with a higher frequency of secondary progressive MS (SPMS) and lower frequency of relapsing remitting MS (RRMS) in infection cases than in controls (*p* < 0.001). In terms of clinical status, clinical duration was longer (*p* = 0.007) and EDSS on admission was higher (*p* < 0.001) in infection cases.

### 3.3 Treatment agents

**Figure 2:**
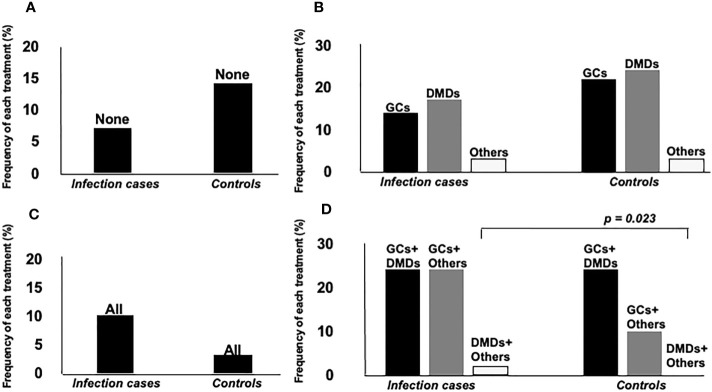
Comparison of frequency of each treatment agent between two groups. Groups were separately analyzed according to the number of treatment agents. **(A)** none, using no drug, **(B)** using only one drug, as GCs, DMDs or others (other kind of immunosuppressant; azathioprine, methotrexate, bucillamine, tacrolimus, cyclosporine), **(C)** all of them, **(D)** using two drugs, as GCs+DMDs, GCs+Others, DMDs+Others. Frequency of GCs+Others users was significantly higher in infection cases than controls.

**Figure 3:**
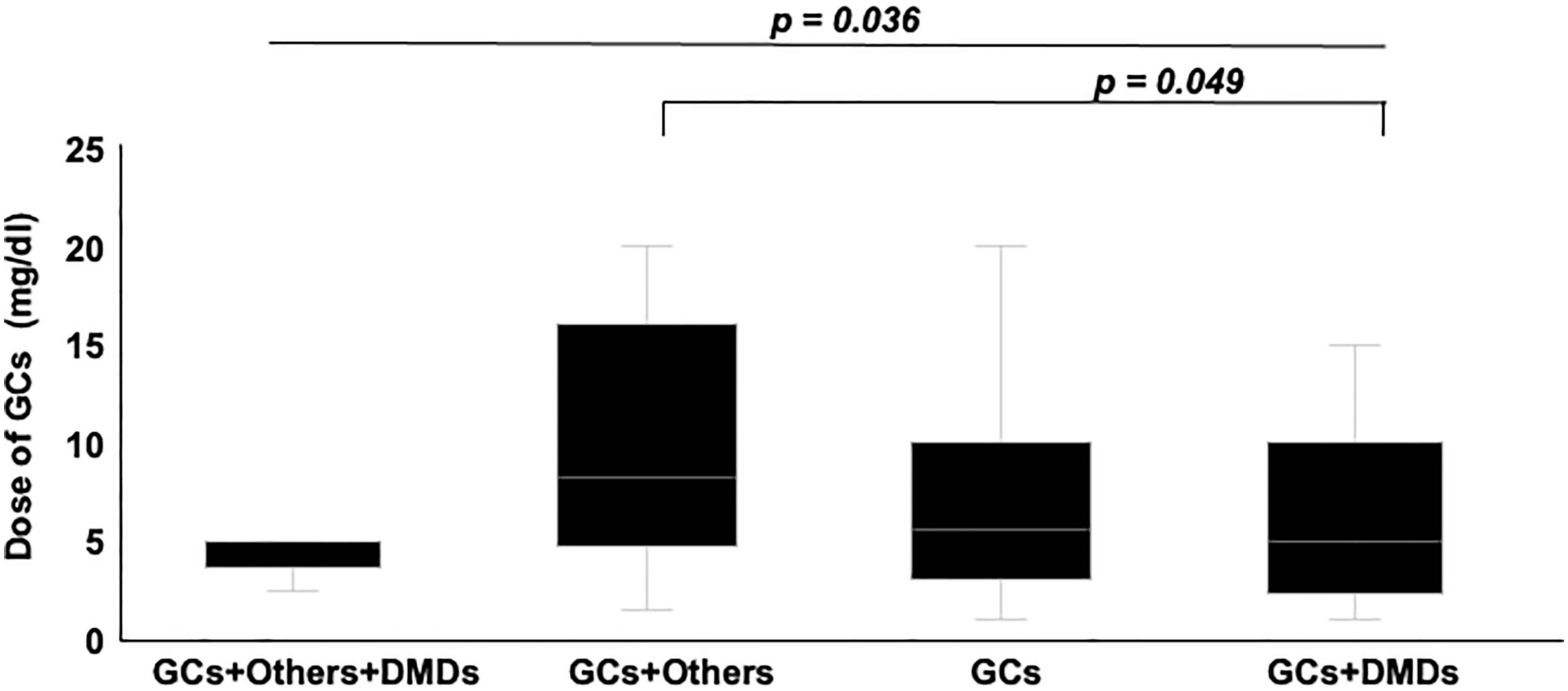
Comparison of GCs dose between groups divided according to treatment subtypes. Groups using Others and GCs showed the highest dose of GCs among groups. *Post hoc* analysis also showed significantly higher dose in groups using others and GCs, compared to group using GCs+DMDs.

Mean dose of GCs and total dose of IVMP within 6 months were higher for infection cases (*p* = 0.005, *p* = 0.006, respectively), but time-equivalent mean dose of IVMP did not differ significantly between groups. Frequency of immunosuppressant agents and DMDs, and duration of DMDs use did not differ significantly between groups. We also divided patients into groups according to how many and what kind of drugs they used. Eight groups were identified: 1) none, using no drug; using only one drug, as 2) GCs, 3) DMDs or 4) other (other kind of immunosuppressant; azathioprine, methotrexate, bucillamine, tacrolimus, cyclosporine); using two drugs, as 5) GCs+DMDs, 6) GCs+Other, 7) DMDs+Other; or 8) use of all three drugs. **Figure 2** shows a comparison of the frequency of each group between IRH cases and controls. The frequency of using GCs+Other was the only significant difference between groups. In the comparison of GCs doses between groups divided according to treatment subtype, subgroups with GCs+Others showed the highest dose of GC among all groups using GCs, and significantly higher than that in GCs+DMDs, after *post-hoc* analysis using Bonferroni methods (**Figure 3**).

### 3.4 Laboratory test results

**Figure 4:**
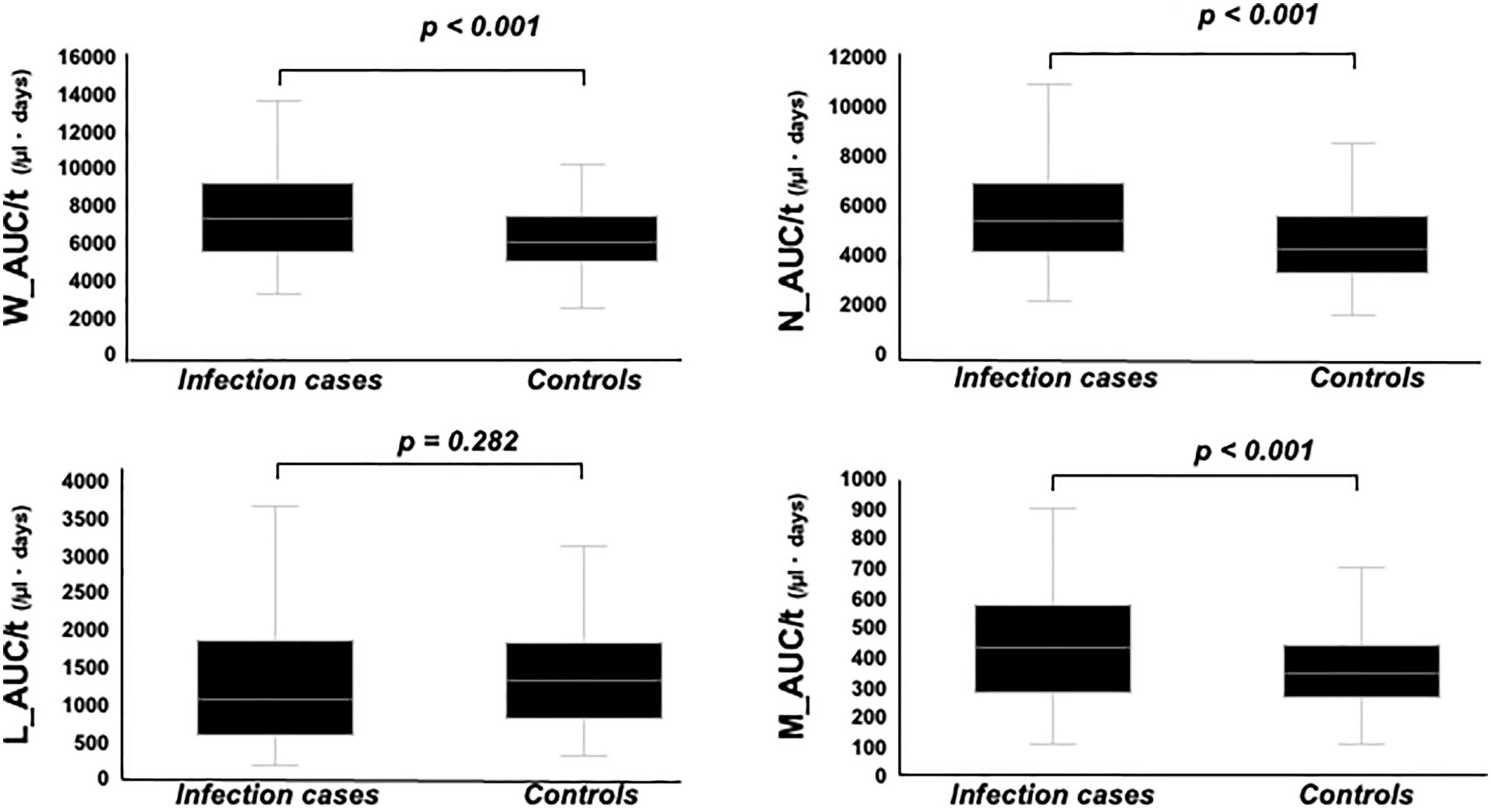
Comparison of W_AUC/t, N_AUC/t, L_AUC/t, and M_AUC/t between infection cases and controls. W_AUC/t, N_AUC/t, and M_AUC/t were significantly higher in infection cases than controls, while L_AUC/t tended to be lower in infection cases than controls, though not statistically significant.

**Figure 5:**
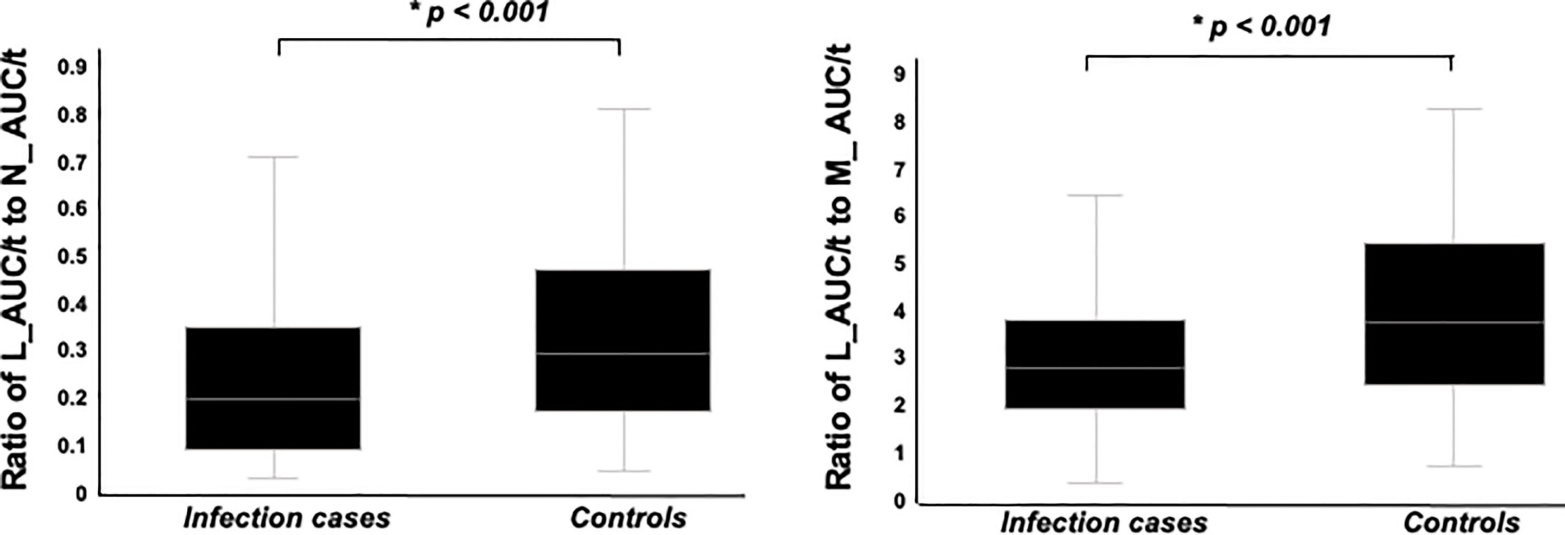
Comparison of ratio of L_AUC/t to N_AUC/t and ratio of L_AUC/t to M_AUC/t between infection cases and controls. Both were significantly lower in infection cases than controls.

W_AUC/t, N_AUC/t, and M_AUC/t were higher in infection cases than in controls (**Figure 4**, *p* < 0.001, each, respectively). On the other hand, L_AUC/t tended to be lower in infection cases than in controls, although no significant differences were identified. To strength the discriminative power of infection, the ratio of L_AUC/t to N_AUC/t or L_AUC/t to M_AUC/t was also analyzed, and both were significantly lower in infection cases than in controls (**Figure 5**, *p* < 0.001 each, respectively).

Notably, both of L_AUC/t to N_AUC/t and L_AUC/t to M_AUC/t were significantly lower in patients using Fingolimod than others (L_AUC/t to M_AUC/t; Fingolimod: median 1.23, range: 0.91-1.40, none of DMDs: median 3.65, range: 2.45-5.68, Interferon-beta 1a/1b: median 3.43, range: 2.77-4.82, Glatiramer acetate: median 3.66, range: 2.74-4.89, Dimethyl fumarate: median 3.50, range: 2.72-4.08, Siponimod fumaric acid: median 1.19, range: 0.96-1.75, Natalizumab: median 4.09, range: 2.93-5.46, Ofatumumab: median 4.10, range: 2.54-4.18, L_AUC/t to N_AUC/t; Fingolimod: median 0.14, range: 0.08-0.15, none of DMDs: median 0.27, range: 0.13-0.46, Interferon-beta 1a/1b: median 0.29, range: 0.20-0.39, Glatiramer acetate: median 0.25, range: 0.19-0.34, Dimethyl fumarate: median 0.31, range: 0.26-0.52, Siponimod fumaric acid: median 0.16, range: 0.12-0.18, Natalizumab: median 0.45, range: 0.35-0.65, Ofatumumab: median 0.19, range: 0.17-0.27).

Serum immunoglobulin G level was analyzed only in cases for which the data had been obtained (n=81; 32 infection cases, 49 controls), and was significantly lower in infection cases, compared to controls (median 733, range: 613-886 mg/dl vs. median 899, range 766-1108 mg/dl, *p* = 0.003).

### 3.5 Predictive factors for infections

**Table 3** shows the results of crude and adjusted conditional logistic regression analyses, which assessed the association between clinical characteristics and infection. Based on the crude OR, patients with SPMS showed a 2.290-times higher risk of severe infection (95%CI 1.290–4.070, *p* = 0.005). Increasing clinical duration and baseline EDSS level by one unit, the risk of serious infection was shown to be 1.040 (95%CI 1.010–1.070, *p* = 0.018) and 1.420 (95%CI 1.220–1.660, *p* < 0.001) times higher, respectively. Also, as mean GC dose increased by one unit, the risk of serious infection was shown to be 1.100 (95%CI 1.040–1.170, *p* = 0.003). On the other hand, as the ratio of L_AUC/t to N_AUC/t and L_AUC/t to M_AUC/t increased by one unit, the risk of serious infection was shown to be 0.237 (95%CI 0.657–0.979, *p* = 0.047) and 0.790 (95%CI 0.666–0.938, *p* = 0.007) times lower. The adjusted OR for risk of serious infection in patients with MS with higher baseline EDSS (OR 1.340, 95%CI 1.070–1.670, *p* = 0.010) and lower ratio of L_AUC/t to M_AUC/t (OR 0.766, 95%CI 0.591–0.993, *p* = 0.046) were statistically significant.

**Table 3:** Monovariate and multivariate regression analysis for infection.

	COR	95% CI	*p*	AOR	95%CI	*p*
Type of MS, SPMS	2.290	1.290-4.070	0.005	0.978	0.435-2.200	0.957
Clinical duration, years	1.040	1.010-1.070	0.018	1.010	0.971-1.050	0.654
Baseline EDSS	1.420	1.220-1.660	<0.005	1.340	1.070-1.670	0.010
GCs dose, mg/d	1.100	1.040-1.170	0.003	1.070	1.000-1.150	0.051
Ratio of L_AUC/t to N_AUC/t	0.237	0.657-0.979	0.003	2.780	0.392-19.800	0.306
Ratio of L_AUC/t to M_AUC/t	0.790	0.666-0.938	0.007	0.766	0.591-0.993	0.010

COR, Crude odds ratio; AOR, Adjusted odds ratio; MS, Multiple sclerosis; SPMS, Secondary progressive multiple sclerosis; EDSS, Expanded Disability Status Scale; PSL, Prednisolone; GCs, Glucocorticoids; AUC, Area under the curve.

### 3.6 Discriminative analysis for infection group and controls

We also conducted receiver operating curve (ROC) analysis of these two variables to extract significant values for discriminating IRH cases. ROC showed optimal cut-offs of 6.0 for baseline EDSS and 3.7 for the ratio of L_AUC/t to M_AUC/t. **Table 4** shows discriminative analyses for infection group and controls by using EDSS and ratio of L_AUC/t to M_AUC/t. Analysis showed optimal thresholds of EDSS ≥ 6.0 to discriminate infection cases and controls with 74.6% sensitivity (95%CI 61.3–84.6%) and 50.8% specificity (95%CI 41.5–60.1%), and ratio of L_AUC/t to M_AUC/t ≤ 3.7 with 69.5% sensitivity (95%CI 56.0–80.5%) and 68.6% specificity (59.4–76.7%). Using either EDSS ≥ 6.0 or ratio of L_AUC/t to M_AUC/t ≤ 3.7 as discriminative variables, sensitivity increased to 88.1% (95%CI 76.5–94.7%), but specificity decreased to 35.6% (95%CI 27.1–45.0%), while using both EDSS ≥ 6.0 and ratio of L_AUC/t to M_AUC/t ≤ 3.7, sensitivity decreased to 55.9% (95%CI 42.5–68.6%), but specificity increased to 83.9% (95%CI 75.7–89.8%).

**Table 4:** Discriminate analysis for infection group and controls by using EDSS or Ratio of L_AUC/t to M_AUC/t.

	EDSS≥6.0	Ratio of L_AUC/t to M_AUC/t ≤ 3.7	Each of EDSS≥6.0 or Ratio of L_AUC/t to M_AUC/t ≤ 3.7	Both of EDSS≥6.0 and Ratio of L_AUC/t to M_AUC/t ≤ 3.7
Infections/Controls	44/58	41/37	52/76	33/19
Sensitivity (95% CI)	74.6 (61.3-84.6)	69.5 (56.0-80.5)	88.1 (76.5-94.7)	55.9 (42.5-68.6)
Specificity (95% CI)	50.8 (41.5-60.1)	68.6 (59.4-76.7)	35.6 (27.1-45.0)	83.9 (75.7-89.8)
Positive predictive value	57.6 (50.0-64.9)	44.1 (36.7-51.7)	72.3 (65.0-78.6)	29.3 (22.9-36.8)
Negative predictive value	42.4 (35.0-50.0)	55.9 (48.3-63.3)	27.7 (21.4-35.0)	70.6 (63.2-77.0)

EDSS, Expanded Disability Status Scale; AUC, Area under the curve.

## 4 Discussion

In this case-control study, we analyzed 59 cases with IRH and 118 controls without serious infection matched for follow-up time and year of cohort entry. Our study showed clinical characteristics of infection in MS patients and the impact of the ratio of L_AUC/t to M_AUC/t as a newly established prognostic factor for IRH.

Among types of infection, UTI was the most common. Given that bladder dysfunction was extremely common in MS and often not appropriately treated, the frequency of UTI appeared reasonable and consistent with previous studies. Specific DMDs-related infections were determined, such as progressive multifocal leukoencephalopathy (PML) with natalizumab (10) or VZV with fingolimod (11). In the present study, one case of PML was detected in a patient receiving natalizumab. VZV infection requiring hospitalization was seen in only one patient, who was using low-dose GC at 4 mg/day, AZA at 25 mg/day, and glatiramer acetate, not fingolimod. The low prevalence of DMDs-related infections may reflect the efficacy of current treatment guidelines.

In comparing the two groups, a higher frequency of SPMS, longer clinical duration, higher baseline EDSS, higher mean GC dose, lower ratio of L_AUC/t to N_AUC/t and lower ratio of L_AUC/t to M_AUC/t were observed for infection cases. Of these, multivariate analysis showed that higher baseline EDSS and lower ratio of L_AUC/t to M_AUC/t were significantly associated with infectious risk.

Many studies have shown MS itself as a risk factor for severe infection because of the uncontrolled immunodeficiency (2–4, 6). Also, some studies have shown that patients with MS in the progressive phase are unable to properly fight infections, reflecting the higher risk of infection-associated hospitalization (2, 3). Higher EDSS is associated with more frequent urological complications, including infection (12), with lower urinary tract dysfunction, catheterization, and greater functional dependence leading to compromised hygiene, potentially increasing the risk of UTI. Respiratory dysfunction is also associated with higher EDSS in MS (13), leading to aspiration pneumonia and lung infection (14). The present study also revealed the association between clinical severity and IRH, consistent with these past studies.

As evaluation at a single time point was not sufficient to assess the risk of infection because of the absence of data on the duration of leukocytopenia, AUC/t, which contains components of both severity and duration of leukocytopenia, offers a more adequate variable for evaluating the risk of infection. This value has been reported as clinically useful for predicting the risk of several infections after allogeneic hematopoietic stem cell transplantation (8, 9), but has not previously been evaluated in MS patients. Also, long-term immunosuppressants may cause decreases in all kinds of leukocytes, but relatively milder decreases in monocytes and neutrocytes, particularly in patients using GCs. Thus, the utility of the ratio of L_ACU/t to M_AUC/t or the ratio of L_ACU/t to N_AUC/t as specific predictors of infection in MS patients seems reasonable.

The prednisolone-equivalent dose of GC did not correlate with serious infection in multivariate analysis. This means that the actual leukocyte count was more important than the dose of GC itself, considering that the severity of leukocytopenia varies even among patients using same dose of GCs.

Also, DMDs use did not differ significantly between infection cases and controls in the present study. Second-generation DMDs such as natalizumab and fingolimod have been found to interfere with immune response and promote infection, compared to placebo or first-generation DMDs such as injectable drugs, interferon-1b and glatiramer acetate (11, 15–17). However, those studies evaluated all infections, and did not distinguish by severity of infection. Recent studies have shown differences in infection between first- and second-generation DMDs users, but no such significant differences among different DMDs (18). In addition, the largest studies conducted recently have compared infection risk among RRMS patients with rituximab, natalizumab, fingolimod, interferon beta, and glatiramer acetate (19), showing that rituximab was associated with the highest rate of serious infections, while other DMDs increased the risk of infection-related physician claims, but not hospitalization, indicating that DMDs may cause minor infections, but not severe infection requiring hospitalization. Given the heterogeneous severity of immune suppression associated with these immunosuppressants, a more meaningful analysis considering variables such as wash-out period or subsequent duration of DMDs is needed.

This study has some limitations. First, the retrospective nature of the data collection raises the possibility of reporting biases and infection events could have been over- or under-reported. Due to COVID-19 virus, public health measures such mask wearing in public, restrictions on leaving the home and social distancing, and frequencies of airborne and droplet-transmitted respiratory infections may have been reduced for a significant proportion of our study. Second, we were unable to account for the medical comorbidities of patients, which may in turn have shown independent associations with risk of infection. The lack of information on several potential confounders such as body mass index, smoking status, or past history of VZV may have influenced the determination of vulnerability to infection. Also, we lacked detailed information on infections related to tissue factors caused by other diseases, such as metallic stents in the urinary tract, or skin barrier vulnerability in skin diseases or hemodialysis shunts in chronic kidney disease. Some comorbidities such as invasive cancer, use of antidepressant and antipsychotic medications, cardiovascular diseases (arrhythmia and major adverse cardiovascular events), diabetes mellitus, and chronic obstructive pulmonary disease are reportedly associated with infection, but none of these were detected in MS patients included in the study. Third, the present results should be limited to cases of serious infection requiring hospitalization. Clinical manifestations might be different with mild infections such as upper respiratory infection or cystitis that prove controllable by oral antibiotics. Fourth, we did not distinguish between community-acquired and in-hospital infections, and only evaluated community-acquired infections, although admission to hospital could represent another factor associated with vulnerability to infection. The results were limited to patients with MS admitted to hospital due to community-acquired infections, and cannot be generalized to in-hospital infections.

Our findings should be considered in risk-benefit assessments of MS therapies, and further monitoring is important using such potential predictive factors.

## 5 Conclusion

Our study showed clinical characteristics of infection in MS patients and the impact of ratio of L_AUC/t to M_AUC/t as new prognostic factors for IRH. Clinicians should pay more attention to laboratory data such as lymphocyte or monocyte counts or ratios rather, directly presenting with individual immunodeficiency, than the kind of drugs to prevent infection as a clinical manifestation.

## Data availability statement

The raw data supporting the conclusions of this article will be made available by the authors, without undue reservation.

## Ethics statement

This study was conducted in accordance with the Declaration of Helsinki, and was approved by the National Center Hospital Ethics Committee (A2019-061).

## Author contributions

JT and TO analyzed the patient data and were major contributors in writing the manuscript. RS, AK, YL, WS, TY, YT helped to draft the manuscript. All authors contributed to the article and approved the submitted version.
